# Sonar image denoising based on clustering and Bayesian sparse coding

**DOI:** 10.1371/journal.pone.0330196

**Published:** 2025-09-02

**Authors:** Chuanxi Xing, Debiao Bao, Tinglong Huang, Yihan Meng

**Affiliations:** 1 School of Electrical and Information Technology, Yunnan Minzu University, Kunming, China; 2 Yunnan Key Laboratory of Unmanned Autonomous System, Yunnan Minzu University, Kunming, China; Whale Wave Technology Inc, CHINA

## Abstract

Side-scan sonar image (SSI) are often affected by a combination of multiplicative speckle noise and additive noise, which degrades image quality and hinders target recognition and scene interpretation. To address this problem, this paper proposes a denoising algorithm that integrates non-local similar block clustering with Bayesian sparse coding. The proposed method leverages cross-scale structural features and noise statistical properties of image patches, and employs a similarity metric based on the Equivalent Number of Looks (ENL) along with an improved K-means clustering algorithm to achieve accurate classification and enhance intra-class noise consistency. Subsequently, a joint training strategy is used to construct dictionaries for each cluster, and Bayesian Orthogonal Matching Pursuit (BOMP) is applied for sparse representation. This enables effective modeling and suppression of mixed noise while preserving structural details. Experimental results demonstrate that the proposed method outperforms several classical approaches in both objective metrics such as PSNR and SSIM, and in visual quality, particularly in preserving target edges and textures under severe noise conditions.

## 1. Introduce

Side Scan Sonar (SSS) technology is a critical tool for underwater detection, extensively applied in seafloor mapping, underwater archaeology, and marine resource exploration. However, the complex and dynamic marine environment severely compromises its imaging quality, with speckle noise posing a predominant challenge. Manifesting as randomly distributed bright and dark granular patterns [1,2], this multiplicative noise degrades image details, deforms target contours, and blurs edges, hindering automated target detection and seabed feature extraction [3]. With the continuous advancement of synthetic aperture sonar (SAS) systems, increasing demands have been placed on denoising algorithms in the post-processing stage [4].

Although a wide range of denoising techniques have been developed, achieving high-fidelity sonar image restoration under severe noise interference remains a challenging task. Traditional methods, such as spatial domain filters [5] and statistical approaches like Wiener filtering [6], rely on local signal statistics to perform adaptive noise suppression. Similarity-based algorithms, including Non-Local Means (NLM) [7] and Block-Matching 3D (BM3D) [8], leverage patch redundancy to enhance denoising performance. In recent years, deep learning models such as DnCNN have shown remarkable success in natural image denoising [9–11]. However, most of these models are designed under the assumption of additive Gaussian noise, which fundamentally differs from the noise characteristics in sonar images, where both multiplicative speckle noise and additive noise commonly coexist [12]. Consequently, these methods often struggle with residual noise or over-smoothing effects, thereby limiting their effectiveness in real-world underwater imaging applications.

Sparse representation has proven to be a highly effective paradigm for image denoising [13], owing to its strong capability to distinguish structured signal components from unstructured noise. Natural images typically exhibit inherent sparsity in an appropriate transform domain, whereas common noise types such as Gaussian or quantization noise do not share this sparse property. This fundamental distinction provides the theoretical foundation for sparse representation-based denoising approaches [14]. Representative methods include Nonlocally Centralized Sparse Representation (NCSR) [15], Learned Simultaneous Sparse Coding (LSSC), and K-Singular Value Decomposition (K-SVD) [16]. Among them, K-SVD constructs an overcomplete dictionary from training data and utilizes Orthogonal Matching Pursuit (OMP) for sparse coding [17]. However, it suffers from two key limitations. First, its block-matching strategy is restricted to local search windows, which limits the ability to capture nonlocal structural redundancy [18]. Second, OMP is vulnerable to pseudo-atom interference, where noise-contaminated correlations lead to the selection of incorrect atoms, significantly degrading performance under multiplicative noise conditions [19].

To address these issues, we propose a novel denoising algorithm that integrates non-local similar block clustering with Bayesian sparse reconstruction. First, an ENL-guided log-distance metric is constructed at the patch level to enhance the accuracy and structural consistency of clustering. Then, cluster-specific dictionaries are trained, and the Bayesian Orthogonal Matching Pursuit (BOMP) algorithm is employed during reconstruction, embedding both structural priors and noise statistics into the sparse representation process. This framework aligns more closely with the statistical nature of speckle noise in sonar images and achieves joint optimization of noise suppression and detail preservation, providing a reliable preprocessing tool for complex sonar imaging tasks.

## 2. Related model construction

### 2.1 Noise model of sonar image

**Fig 1:**
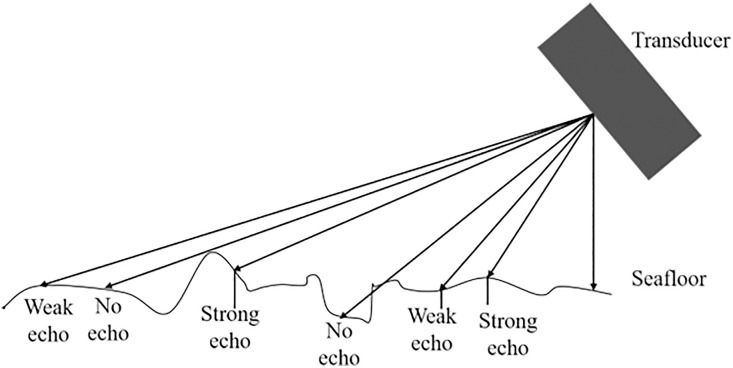
Side-scan sonar imaging principle.

This study focuses explicitly on speckle noise induced by seabed reverberation in SSI. From the perspective of imaging physics, SSS generates images by emitting narrow-beam acoustic pulses toward the seafloor and capturing backscattered signals from both the seafloor surface and sub-surface layers. During this process, as acoustic waves interact with the rough seabed interface, coherent echoes produced by numerous micro-scale scatterers undergo random constructive and destructive interference upon reaching the receiving array. This mechanism, illustrated in Fig 1. Side-scan sonar imaging principle, elucidates speckle noise’s physical origins and statistical properties [20], revealing its spatially varying intensity distribution and formation principles.

This underlying physical mechanism inherently results in multiplicative speckle noise in sonar images. Such noise exhibits signal-dependent statistical properties, which fundamentally distinguish it from additive noise models. The speckle noise observed in sonar imaging shares similar characteristics with that encountered in synthetic aperture radar (SAR) systems [21,22]. In these cases, the noise model can be mathematically described as follows:


$$\begin{array}{c} f=un_{mul} \end{array}$$ (1)

Where f is SSI corrupted by multiplicative speckle noise, u is the original noise-free sonar image, and $n_{mul}$ is the multiplicative speckle noise, which follows a Rayleigh distribution. The randomness in the spatial distribution of seabed scatterers manifests in the statistical properties of the noise component. The probability density function of the noise term, $p_{x}(x)$,is given by:


$$\begin{array}{c} p_{x}(x)=\left\{ \;\;\;\begin{array}{c} 0\;x<1\\ \frac{x}{\sigma^{2}}\exp\left(-\frac{x^{2}}{2\sigma^{2}}\right)\;x\geq 1 \end{array}\right.\; \end{array}$$ (2)

According to the findings presented in the literature [5], the noise characteristics in real-world SSS systems are non-stationary. In addition to the previously discussed multiplicative noise caused by scattering interference, additive noise components arise from various sources, including thermal noise generated by electronic system components and ambient noise originating from the underwater environment. Considering these factors, the composite noise model for sonar images can be expressed as:


$$\begin{array}{c} f=u+un_{mul}+n_{add} \end{array}$$ (3)

In this model, $n_{add}$ is the additive noise that satisfies the Gaussian distribution $𝒩(0,\;\sigma^{2})$, and its standard deviation is σ.

### 2.2 Sparse representation model construction

A sparse representation framework for phased noise suppression is developed based on the composite noise model presented in Equation (3). The observed noisy image can be modeled as follows:


$$\begin{array}{c} Y=X\left(E+N_{mul}\right)+N_{add} \end{array}$$ (4)

In this equation, Y is the noisy observed image, $X\in {\mathbb{R}}^{N\times N}$ denotes the original clean image, $N_{mul}$ is the multiplicative speckle noise matrix following a Rayleigh distribution, and $N_{add}$ represents the additive noise.

According to sparse representation theory, additive noise in sonar images can be effectively removed, resulting in a refined sonar noise model after eliminating additive Gaussian noise.


$$\begin{array}{c} Y={\hat{X}}_{multi}+N_{add} \end{array}$$ (5)

$$\begin{array}{c} {\hat{X}}_{multi}=X\left(E+N_{mul}\right) \end{array}$$ (6)

The signal ${\hat{X}}_{multi}$ represents the reconstructed image after additive noise removal.

To further suppress the multiplicative noise present in sonar images, a homomorphic trans formation is applied to ${\hat{X}}_{multi}$, converting the multiplicative noise into an additive form. Specifically, the logarithmic operation is performed on Equation (13):


$$\begin{array}{c} \log{\hat{X}}_{multi}=\log\left(X\left(E+N_{mul}\right)\right)=\log(X)+\log\left(E+N_{mul}\right) \end{array}$$ (7)

Assume that $\tilde{X}=\log{\hat{X}}_{multi}$, $X_{\log}=\log(X)$, ${\hat{N}}_{mul}=log\left(E+N_{mul}\right)$, then equation (14) is equal to


$$\begin{array}{c} \tilde{X}=X_{\log}+{\hat{Z}}_{s} \end{array}$$ (8)

Here, the multiplicative noise in ${\hat{X}}_{multi}$ is transformed into additive noise$\;{\hat{Z}}_{s}$ through the logarithmic operation. Using a DCT-based sparse representation model, the signal $X_{\log}$ can be reconstructed from $\tilde{X}$. Finally, the denoised signal X is recovered by applying the inverse logarithmic transformation to $X_{\log}$, and the reconstructed sonar image is obtained by reshaping the result.

## 3. Overall algorithm design

**Fig 2:**
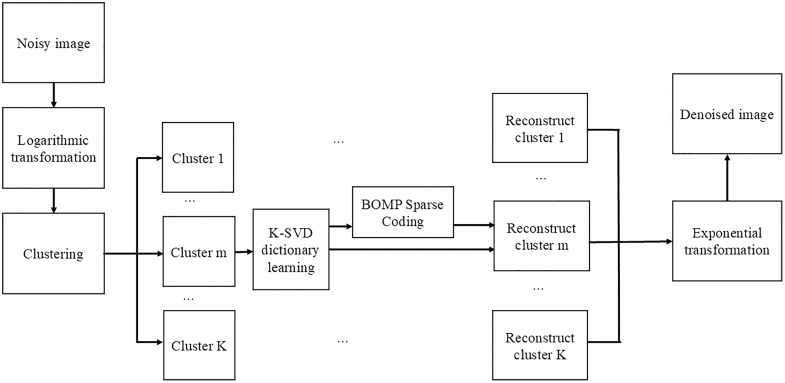
Flow chart of image-denoising algorithm.

Fig 2 presents the overall workflow of the proposed sonar image denoising algorithm. The process begins with a logarithmic transformation of the noisy sonar image, which converts multiplicative noise into additive noise. This is followed by non-local clustering, where similar image patches are grouped into K clusters based on an ENL-guided similarity metric. For each cluster, a custom dictionary is trained using the K-SVD algorithm, capturing shared structural features. Then, BOMP is applied to perform sparse coding, producing high-fidelity reconstructions for each cluster. Finally, the reconstructed patches are aggregated, and an exponential transformation is applied to map the image back to the intensity domin, yielding the final denoised result.

### 3.1 Image clustering

Although the K-means algorithm is computationally efficient and widely used for general data clustering, it faces significant limitations when applied to SSI. These limitations arise from the incompatibility between the algorithm’s fundamental assumptions and the signal-dependent multiplicative nature of speckle noise found in SSS systems. Unlike additive noise models, where noise components are statistically independent of the signal intensity, speckle noise exhibits an amplitude correlation with the underlying backscatter signal. This characteristic violates the independent and identically distributed noise assumption central to conventional Euclidean distance metrics. As a result, this mismatch introduces systematic clustering biases, including overemphasis on noise-driven intensity variations, reduced sensitivity to actual seabed textures, and incorrect classification of boundary transitions. The standard Euclidean distance formulation:


$$\begin{array}{c} d\left(x_{c}\;,\;x_{n}\right)={\left\|x_{c}-x_{n}\right\|}_{2} \end{array}$$ (9)

The conventional Euclidean distance metric presents two key limitations in the presence of mixed additive and multiplicative noise: it tends to amplify intra-class variations in regions with high signal intensity, and it fails to accurately capture the true boundaries between distinct signal regions. These dual distortions significantly compromise the topological fidelity of clustering outcomes. To address this issue, we introduce a distance metric incorporating the ENL, which is specifically adapted to the characteristics of composite noise in sonar imagery [18], and is formulated as:


$$\begin{array}{c} d\left(x_{c}\;,\;x_{n}\right)=\frac{1}{L}\sum_{j}\left(\frac{x_{c}(j)}{x_{n}(j)}+\frac{x_{n}(j)}{x_{c}(j)}\right) \end{array}$$ (10)

L represents the equivalent number of looks; j is the index that traverses all pixels in the image block.


$$\begin{array}{c} L=\frac{\mu^{2}}{\sigma^{2}} \end{array}$$ (11)

In this formulation, μ represents the mean intensity of the image block, and $\sigma^{2}$ denotes its variance. The Equivalent Number of Looks is a critical parameter for quantifying noise levels through statistical characterization. A higher L value correlates with reduced noise contamination, enabling more precise similarity assessments between image patches. Conversely, a lower L value indicates elevated noise intensity, under which the enhanced distance metric adaptively modulates its computational strategy following local noise statistics. This adaptation mechanism significantly improves the robustness of clustering operations and the reliability of resultant classifications in noisy environments.

### 3.2 BOMP sparse coding

#### 3.2.1 Sparse representation under the Bayesian framework.

Parse representation is a fundamental concept in signal processing, centered around representing signals with as few non-zero coefficients as possible. This approach reduces data dimensionality, enhances computational efficiency, and improves models’ robustness and generalization capability. The sparse signal recovery problem can be formulated within the Bayesian framework as a Maximum A Posteriori (MAP) estimation problem. The most probable sparse coefficient vector can be determined by maximizing the posterior probability of the sparse coefficients, enabling the reconstruction of the original signal.

The known noise model is represented as Equation (15) in the model described above. Under the Bayesian framework, assuming the sparse coefficients α follow an independent Gaussian prior distribution, the MAP estimation objective function can be expressed as:


$$\begin{array}{c} \hat{\alpha }=\mathrm{\backslash arg}\min_{\alpha }P\left(x|y\right)=\mathrm{\backslash arg}\min_{\alpha }\frac{P\left(y|x\right)P(x)}{P(y)} \end{array}$$ (12)

According to Bayes’ theorem, P(y) serves as a normalization constant and can thus be disregarded. Assuming a Gaussian distribution, this optimization problem can be reformulated as the minimization of the following objective function:


$$\begin{array}{c} \hat{\alpha }=\mathrm{\backslash arg}\min_{\alpha }\left[{\left\|y-D\alpha \right\|}_{2}^{2}+\lambda {\left\|\alpha \right\|}_{2}^{2}\right] \end{array}$$ (13)

Where $\lambda =\frac{\sigma_{\epsilon }^{2}}{\sigma_{x}^{2}}$ is the adaptive regularization parameter. $\sigma_{\epsilon }^{2}$ is the noise variance, and $\sigma_{x}^{2}$ is the signal prior variance.

#### 3.2.2. Principal optimization of the BOMP algorithm.

Unlike the traditional OMP, which selects atoms based purely on the maximum inner product with the residual, the BOMP method incorporates prior knowledge of both the signal and noise distributions. Specifically, BOMP [23] selects atoms based on a cost function derived from MAP estimation, which includes terms for reconstruction error and model complexity. This probabilistic framework enhances robustness in noisy environments—particularly for multiplicative noise common in sonar images—by dynamically estimating noise variance and avoiding overfitting. As a result, BOMP achieves more stable sparse reconstructions compared to OMP.

The BOMP algorithm improves upon traditional OMP by incorporating not only the residual energy but also sparse coefficient variation and model complexity into the atom selection criterion. To enhance clarity, we decompose the atom selection process into two parts: one for estimating the energy change caused by selecting a candidate atom, and another for penalizing sparsity and complexity.

The first part estimates the change in reconstruction error if atom $D_{i}$ is added to the support set:


$$\begin{array}{c} \Delta E_{i}={\left\|r_{t-1}\right\|}^{2}+2D_{i}^{T}r_{t-1}\left({\hat{\alpha }}_{i}-{\tilde{\alpha }}_{i}\right)+{\left({\hat{\alpha }}_{i}-{\tilde{\alpha }}_{i}\right)}^{2}{\left\|D_{i}\right\|}^{2} \end{array}$$ (14)

Where $D_{i}$ represents the i atom in the dictionary, ${\hat{\alpha }}_{i}$ is the currently estimated sparse coefficient, ${\tilde{\alpha }}_{i}$ is the sparse estimate from the previous iteration. This expression reflects how the residual is expected to change based on the correlation between the residual and the atom, as well as the difference in estimated sparse coefficients.

The complete cost function used to determine the optimal atom is:


$$\begin{array}{c} i^{*}=arg\min_{i}\sqrt{\Delta E_{i}}-\frac{\sigma_{\epsilon }^{2}}{\sigma_{x}^{2}}{\tilde{\alpha }}_{i}^{2}+\sigma_{\epsilon }^{2}\Delta S \end{array}$$ (15)

Where $\Delta S=I(i\notin S_{t-1})$ represents the complexity penalty for adding new atoms, helping to prevent overfitting.$\sigma_{\epsilon }^{2}$ is the dynamically estimated noise variance, and its expression is:


$$\begin{array}{c} \sigma_{\epsilon }^{2(t)}=\frac{{\left\|r_{t}\right\|}_{2}^{2}}{N-\left|S_{t}\right|} \end{array}$$ (16)

The formula, denotes the residual at the t iteration, N is the dimension of the observed signal, and $\left|S_{t}\right|$ represents the number of basis vectors in the current support set.

This formula leverages the current residual energy to provide a real-time varying noise levels, thereby improving the algorithm’s adaptability to varying noise levels. By combining the strengths of Bayesian learning and OMP, BOMP demonstrates enhanced robustness in handling multiplicative noise signals. It effectively mitigates the impact of noise during the sparse representation process, resulting in improved signal reconstruction quality.

#### 3.2.3. Parameter update.

The BOMP algorithm enhances the support set updating process and coefficient estimation through a Bayesian regularization strategy. Its core concept incorporates prior information to improve numerical stability and generalization performance. Unlike traditional OMP methods that update the support set solely based on the atom most correlated with the current residual, BOMP integrates Bayesian learning into the atom selection process, developing a more robust update mechanism. During each iteration, the optimal atom index $i^{*}$ is determined according to the Bayesian atom selection criterion defined in Equation (19), followed by subsequent support set updates.


$$\begin{array}{c} S_{t}=S_{t-1}⋃\left\{i^{*}\right\}\; \end{array}$$ (17)

Where $S_{t}$ is the support set at the current iteration, and $i^{*}$ denotes the index of the optimal dictionary atom selected in this round.

After updating the support set, BOMP needs to estimate the sparse coefficients. Unlike OMP, which directly solves the least squares problem, BOMP uses Bayesian posterior mean estimation to optimize the coefficient calculation process. For the current support set $S_{t}$, the coefficient estimation can be calculated using the following formula:


$$\begin{array}{c} {\hat{\alpha }}_{S_{t}}=E\left[\alpha_{S_{t}}|y,S_{t}\right]={\left(D_{S_{t}}^{T}D_{S_{t}}+\sigma_{\epsilon }^{2}\sum_{x}^{-1}\right)}^{-1}D_{S_{t}}^{T}y \end{array}$$ (18)

Where $D_{S_{t}}$ is the sub-dictionary matrix corresponding to the current support set, and $\sum_{x}=\sigma_{x}^{2}I$ is the prior covariance matrix.

The BOMP iteration is terminated when either the number of selected atoms reaches a predefined maximum $T_{\max}$, or the residual energy$\;{\left\|r_{t}\right\|}_{2}^{2}$ falls below a threshold.

## 4. Experiments and analysis

### 4.1 Experimental parameters and indicators

The experimental validation follows a structured evaluation framework, employing a multi-level comparative analysis to assess the effectiveness of the improved algorithm. The design adopts a progressive approach, transitioning from controlled conditions to real-world applications. In the first stage, standard seafloor terrain images are used to generate test samples by introducing simulated sonar speckle and additive noise, providing benchmark data with known ground truth for precise quantitative evaluation. In the second stage, the algorithm is applied to authentic SSI to comprehensively evaluate its adaptability and robustness under complex noise conditions. All sonar images used in this study are sourced from the publicly available SeabedObject-SSS dataset.

The standardized preprocessing procedure was implemented to ensure the reliability and comparability of the experimental results. All test images were first resized to a uniform resolution of 400 × 400 pixels and converted to grayscale format. To simulate realistic imaging conditions, Gaussian white noise with a standard deviation of 10 and multiplicative speckle noise with a variance of 0.1 were added to each image. Consistent parameter settings were applied across all experiments, including an 8 × 8 window size, a dictionary comprising 256 atoms, 10 iterations for the *K*-SVD algorithm, and noise variance parameters set to $\sigma_{\epsilon }^{2}$=0.01 and $\sigma_{x}^{2}$=1, in accordance with [23].

The comprehensive evaluation framework was established to assess denoising performance across multiple dimensions. Subjective assessment emphasized visual quality, focusing on key aspects such as noise suppression, edge preservation, and detail retention. For objective evaluation, four representative quantitative metrics were selected: The Structural Similarity Index (SSIM) evaluates the preservation of structural information and aligns closely with human visual perception. The Peak Signal-to-Noise Ratio (PSNR) indicates the overall similarity between the denoised and original images. The Mean Squared Error (MSE) measures pixel-wise reconstruction accuracy. The Equivalent Number of Looks-based Performance Index (EPI) assesses background homogeneity and speckle suppression, serving as a complementary metric for evaluating denoising performance. Collectively, these metrics provide a well-rounded and rigorous assessment of image quality.

The formula for calculating SSIM is as follows:


$$\begin{array}{c} SSIM(X,Y)=\frac{\left(2\mu_{X}\mu_{Y}+C_{1}\right)\left(2\sigma_{XY}+C_{2}\right)}{\left({\mu_{X}}^{2}+{\mu_{Y}}^{2}+C_{1}\right)\left({\sigma_{X}}^{2}+{\sigma_{Y}}^{2}+C_{2}\right)} \end{array}$$ (19)

Where $\mu_{X}$ and $\mu_{Y}$ denote the mean values of images X and Y, respectively, while ${\sigma_{X}}^{2}$ and ${\sigma_{Y}}^{2}$ represent the corresponding variances of images X and Y. and $\sigma_{XY}$ is the covariance corresponding to images X and Y.

The calculation formula for PSNR is as follows:


$$\begin{array}{c} PSNR=10\mathrm{lg}\frac{255^{2}}{MSE} \end{array}$$ (20)

$$\begin{array}{c} MSE=\frac{1}{MN}\sum_{i=1}^{M}\sum_{j=1}^{N}{\left(f(i,j)-\hat{f}(i,j)\right)}^{2} \end{array}$$ (21)

Where M and N represent the dimensions of the image, f and $\hat{f}$ represent the original image and the reconstructed image, respectively.

### 4.2 Experimental

**Fig 3:**
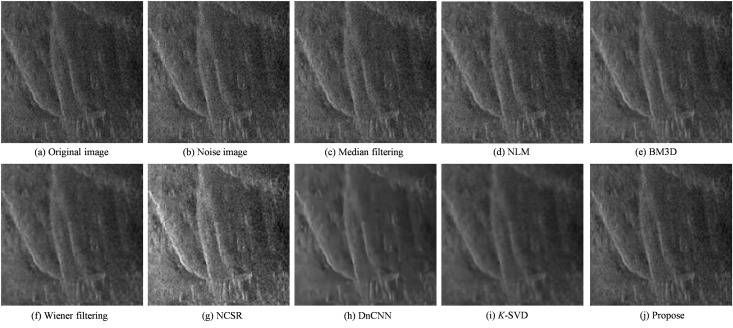
Figures of Results for Various Algorithms.

To simulate typical multiplicative noise commonly encountered in sonar imaging scenarios, a composite noise model was applied by combining Gaussian noise and Rayleigh noise, resulting in the degraded image shown in Fig 3(b). This synthesized noise setup closely mimics the statistical characteristics of real-world sonar image distortions, thereby providing a controlled environment for evaluating denoising algorithms. Subsequently, various representative denoising techniques were employed to restore the corrupted image, including Median Filtering, NLM, BM3D, NCSR, Wiener Filtering, DnCNN, *K*-SVD, and the proposed method.

The denoised results obtained using these methods are shown in Fig 3(c)–(j). Quantitative evaluation results of these methods on Fig 3(b) are summarized in Table 1, based on metrics such as SSIM、PSNR、MSE and EPI, providing a comprehensive assessment of each algorithm’s performance.

**Table 1:** Results of various algorithms.

Method	SSIM	PSNR	MSE	EPI
Median filtering	0.8576	28.728	87.1533	0.7451
NLM	0.9041	28.9743	82.3481	0.8593
BM3D	0.9103	29.0598	80.7427	0.9431
Wiener filtering	0.8519	28.6128	89.4959	0.4629
NCSR	0.9031	29.0621	80.7003	0.8599
DnCNN	0.7807	27.8437	106.8351	0.7894
*K*-SVD	0.8391	31.3695	47.4384	0.5297
Propose	0.9115	33.7257	27.5746	0.9787

A comprehensive comparison of denoising performance can be conducted by systematically analyzing the processing results of various algorithms presented in Fig 3 and referencing the quantitative evaluation metrics summarized in Table 1. While simple and computationally efficient, traditional median filtering demonstrates limited effectiveness in addressing complex speckle noise. The NLM and BM3D algorithms, although widely recognized for their performance in natural image denoising, exhibit inadequate noise suppression when applied to side-scan sonar images affected by severe multiplicative noise. As a classic adaptive technique, Wiener filtering effectively suppresses noise but often compromises image details—particularly under intense speckle noise—leading to noticeable blurring. Although the NCSR algorithm reduce noise to a certain extent, as observed in Fig 3(g), the resulting image still suffers from significant detail blurring, especially around object edges and texture regions. This results in weakened structural information and reduced overall image contrast.

Additionally, residual granular noise in the background indicates the algorithm’s limited capacity to handle complex multiplicative noise. The deep learning-based method DnCNN demonstrates superior denoising performance, effectively suppressing most noise while preserving image structure. However, some areas in the output exhibit slight over-smoothing, which may obscure subtle features, posing challenges for identifying small targets in sonar imagery. As a representative of sparse representation methods, *K*-SVD offers particular advantages in maintaining edges and textures. Nonetheless, its performance is sensitive to training data and parameter settings under complex noise distributions, and slight residual noise remains in the final image.

In comparison, the proposed method demonstrates superior visual quality and structural restoration performance over the other benchmark algorithms. It effectively suppresses complex multiplicative speckle noise and better preserves target contours and background structural details, exhibiting higher contrast and clarity in multiple key regions. As shown in Table 1, the proposed method achieves the best results regarding SSIM, PSNR, and MSE, indicating its excellence in maintaining structural similarity, improving signal-to-noise ratio, and minimizing pixel-wise reconstruction error. These results confirm the effectiveness of the method for denoising SSI.

**Fig 4:**
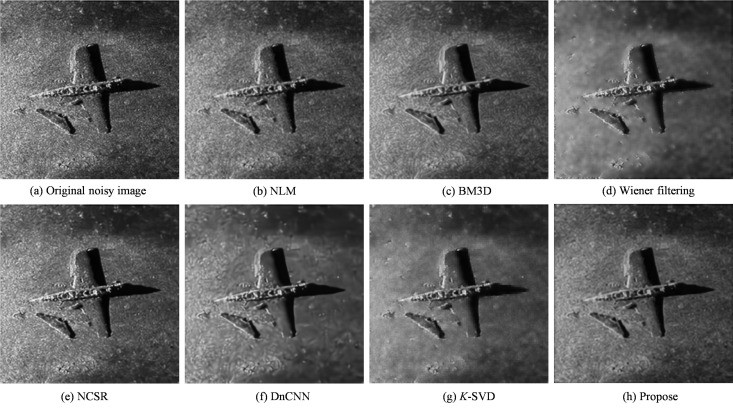
Underwater plane crash denoising experiment.

**Fig 5:**
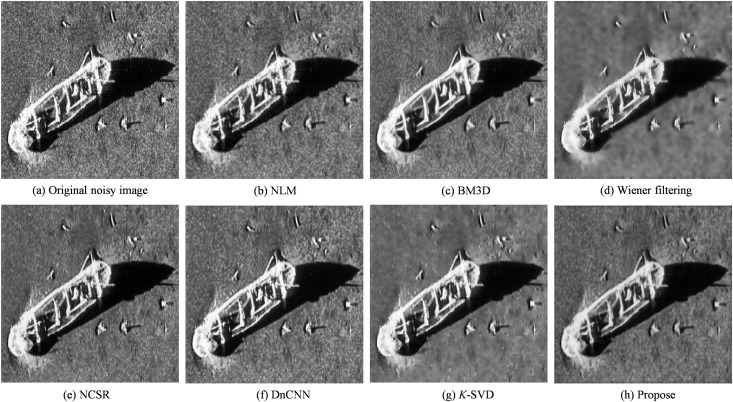
Underwater shipwreck denoising experiment.

Two comparative experiments were conducted to further validate the generalization ability and practical adaptability of the proposed approach. The first experiment used an image of a sunken airplane on the seafloor, as shown in Fig 4. While the second experiment was based on an image of an underwater shipwreck, as shown in Fig 5. These two experiments employed real sonar data with different noise characteristics, providing a focused and representative evaluation of each denoising method’s performance under complex underwater conditions.

Following the denoising experiment on the sonar image of the underwater aircraft, the denoising process was further applied to a sonar image of an underwater shipwreck. The corresponding experimental results are presented in Fig 5.

Upon completing the denoising experiment on the underwater shipwreck image, it was found that although NCSR and DnCNN performs well under simulated noise conditions, it fails to suppress speckle noise prevalent in the shipwreck background adequately. While Wiener filtering can mitigate speckle noise to a certain extent, it often introduces visible artifacts that compromise overall image quality. In contrast, the proposed algorithm exhibits strong adaptability and robustness, effectively removing both types of speckle noise while preserving critical structural information and fine image details. These results underscore the algorithm’s superior capability in addressing complex noise patterns in authentic sonar imagery, ensuring enhanced target visibility and improved background clarity.

### 4.3 Analysis

In order to systematically evaluate the performance of various denoising algorithms, the experiment involved a comprehensive comparison of classical and state-of-the-art techniques, including the NLM algorithm, BM3D, NCSR, traditional Wiener filtering, and the deep learning-based DnCNN model. Despite their strong performance in natural image denoising, methods such as NLM and BM3D show apparent limitations when applied to SSI, particularly in effectively suppressing speckle noise. While Wiener filtering demonstrates a particular capability in noise reduction, it often results in reduced image contrast and a pronounced blurring effect, giving the image an unnatural, grayish tone. The NCSR and DnCNN model performance significantly declines when processing high-intensity speckle noise in SSI.

The proposed denoising algorithm demonstrates significant advantages in handling complex noise conditions. By integrating cross-scale features of image patches with the statistical characteristics of both additive and multiplicative noise, the method achieves accurate noise modeling and effective suppression of Gaussian and speckle noise simultaneously. Experimental evaluations on SSI of underwater aircraft and shipwrecks confirm that the algorithm excels in noise removal and preserves essential structural details, including edges and textures. Quantitative results show substantial improvements in objective metrics such as PSNR and SSIM. At the same time, the enhanced visual clarity and fidelity of the denoised images provide a more reliable basis for downstream tasks such as underwater target detection and recognition.

## 5. Conclusion

To address the issue of combined multiplicative speckle noise and additive noise commonly present in side-scan sonar images, this paper proposes an adaptive denoising algorithm that integrates non-local similarity modeling with Bayesian sparse representation. The algorithm first applies a logarithmic transformation to convert multiplicative noise into an additive form. Then, a multi-scale non-local block matching strategy is employed, followed by an improved K-means clustering algorithm guided by the ENL, which enhances both the accuracy of block classification and intra-class noise consistency. Based on the clustered blocks, adaptive dictionaries are learned, and BOMP is used for sparse reconstruction, enabling effective suppression of complex noise while preserving structural fidelity. Experimental results demonstrate that the proposed method outperforms traditional approaches such as Wiener filtering, median filtering, and conventional sparse representation-based denoising, particularly under high-noise conditions, where it maintains superior detail preservation and reconstruction stability.

Despite its strong denoising performance, the proposed method involves multi-stage feature extraction and iterative optimization, which results in relatively high computational complexity and may limit its applicability in real-time scenarios. Furthermore, some degree of local blurring may still occur in highly textured regions. Future work will focus on algorithm simplification, automated parameter tuning, and enhanced modeling of high-frequency details.

In summary, the proposed method effectively suppresses composite noise while preserving critical structural and textural features, providing a reliable preprocessing foundation for downstream tasks such as sonar image target detection, recognition, and scene interpretation.
